# Supplementary material for: CRISPR/Cas9-mediated gene targeting in Arabidopsis using sequential transformation
Source: Nat Commun. 2018 May 17;9:1967. doi: 10.1038/s41467-018-04416-0 (PMC5958078; doi:10.1038/s41467-018-04416-0)
Supplement: Supplementary file 1 — Supplementary Information [file 41467_2018_4416_MOESM1_ESM.docx]

**CRISPR/Cas9-mediated gene targeting in *Arabidopsis* using sequential transformation**

Miki *et al.*

**Supplementary Figure 1. Gene targeting using all-in-one constructs. a,** Schematic showing the all-in-one gene targeting CRISPR/Cas9 construct that contains: (i) Cas9 driven by the CaMV 35S promoter (35Spro::Cas9), (ii) an sgRNA, driven by the AtU6 promoter, and (iii) a donor DNA fragment for in-frame *GPF* knock-in. The transgenic plants were selected by hygromycin resistance (35Spro::HPT). The lower schematic represents part of the targeted *ROS1* locus and its sgRNA. **b,** Screening of *ROS1*-*GFP* gene targeting in T1 transgenic lines. Upper panel, PCR. Lower panel, Southern blotting. Although GT positive was detected by PCR in the #27, Southern blotting indicates this is not heritabe GT. **c,** Schematic showing the endogenous *GL2* target locus and its donor sequence. Red arrows indicate primers used for screening PCR. Blue triangles indicate HindIII sites, and the horizontal line indicates the probe for Southern blot hybridization. **d,** **e,** PCR for T1 and bulk T2 screening for DD45 promoter and YAO promoter driven Cas9 *GL2*-*GFP* gene targeting, respectively. **f,** Southern blotting for T1 plants. **g-i,** Southern blotting for individual T2 plants. DD45pro::Cas9 *GL2*-*GFP* T2-18 (**g**) and T2-20 (**h**) were from **d**, and YAOpro::Cas9 *GL2*-*GFP* T2-33 (**i**) was from **e**. In the Southern blotting, arrows indicate the size of endogenous *ROS1* or *GL2*, and arrowheads indicate the expected size of *ROS1*-*GFP* or *GL2*-*GFP*.

**Supplementary Figure 2. Detailed information on the targeted *ROS1* locus and donor construct. a,** Schematic showing donor construct and endogenous *ROS1* target locus. **b,** Detailed schematic with sequences of sgRNA, donor and *ROS1* target site. Green bases indicate the stop codon of *ROS1*, and orange bases denote the PAM sequence of sgRNA. The blue triangles and square indicate HindIII restriction enzyme sites. Horizontal line indicates the probe for Southern blot hybridization. Arrows and arrow heads represent PCR primers, which are described in **c**.

**Supplementary Figure 3. Detailed information on the targeted *DME* locus and donor construct. a, b,** Schematics showing donor transgene, and the *DME* target locus for in-frame *DME*-*GFP* (**a**) and *GFP*-*DME* (**b**) gene targeting, respectively. The magenta and green letters indicate start and stop codons of *DME*, and orange denotes PAM sequence of sgRNAs, respectively. The green highlights indicate DNA bases for silent mutation in the donor sequence. The blue triangles indicate restriction enzyme sites, and horizontal lines indicate probes for Southern blot hybridization. Arrows represent primers for detection of gene targeting (GT), which are described in **c**.

**Supplementary Figure 4. Sequence confirmation of the *GFP* or *Luc* knock-in flanking regions.** Sequencing results of border regions are shown for *ROS1*-*GFP* and *ROS1*-*Luc* (**a**), *DME*-*GFP* (**b**), and *GFP*-*DME* (**c**).

**Supplementary Figure 5. Sequence results of *GFP* or *Luc* integration flanking regions.** Sequencing results are shown for *ROS1*-*GFP* and *ROS1*-*Luc* (**a**), *DME*-*GFP* (**b**), and *GFP*-*DME* (**c**). Orange characters in sgRNA indicate PAM sequence, green characters indicate stop codon, magenta characters indicate start codon, and green highlights indicate silent mutations.

**Supplementary Figure 6. Sequences showing amino acid substitution in the *DME* target locus. a**, Schematic showing the endogenous *DME* genomic sequence and HDR donor for amino acid substitution. Blue square indicates the site of amino acid substitution. Southern blotting was performed by using the restriction enzymes PvuI and SacI, and the probe indicated by the horizontal line. Red arrows indicate PCR primers used for genotyping. **b, c**, WT and amino acid substitution donor sequences of *DME*. Orange characters in sgRNA indicate PAM sequence, blue highlights indicate amino acid substitutions, green highlights indicate silent mutations, and horizontal lines indicate restriction enzyme sites used for screening and Southern blotting.

**Supplementary Figure 7. DNA methylation analysis of the *ROS1*-*GFP* target locus. a**, Schematic showing *ROS1*-*GFP* donor and endogenous *ROS1* locus. The two horizontal lines indicate the regions analyzed by individual locus bisulfite sequencing. **b, c**, Individual locus bisulfite sequencing results. One Col-0 and two independent T4 homozygous *ROS1*-*GFP* plants were analyzed. Purple, total C methylation; red, CG methylation; blue, CHG methylation; green, CHH methylation. At least 27 clones were sequenced for each sample for the bisulfite sequencing analysis. Sequencing results were analyzed using Kismeth.

**Supplementary Figure 8. T-DNA copy numbers do not contribute to efficient GT.** T2 bulk DNA samples were analyzed to determine the donor and Cas9 transgene copy numbers by qPCR. **a,** *DME*-*GFP*, **b,** *GFP-DME*, **c,** *DME*-*P1633A*, respectively. The analyzed data represents relative value from *Act7*. The red bar and arrows indicate GT positive samples.

**Supplementary Figure 9. Data distribution by dot-plots. a**, Figure 1f, qRT-PCR for *ROS1*. **b, c**, Figure 1h, qChop-PCR for At1g26400 and At1g03890 loci.

**Supplementary Figure 10. Full-size images of the most important Southern blots. a**, Figure 2c, Southern blotting for *ROS1*-*GFP* T2 individual plants. **b,** Figure 2d, Southern blotting for *ROS1*-*GFP* T3. **c**, Figure 3c, Southern blotting for *DME*-*GFP* T2 individual plants. **d**, Figure 3e, Southern blotting for *DME*-*GFP* T3.

**Supplementary Table 1. GT efficiency for the *ROS1* locus using the 35Spro::Cas9 all-in-one construct**

| **donor** | **T1** | **T2** | |
| --- | --- | --- | --- |
|  |  | **bulk** | **Individual plants** |
| *GFP* with gRNA | 2/30 | 1/18 | 0/21 |
| *GFP* without gRNA | 0/29 | N.D. | N.D. |
| *Luc* with gRNA | 0/12 | 1/8 | 0/20 |
| *Luc* without gRNA | 0/24 | N.D. | N.D. |

For T2 bulk analysis, the corresponding T1 plants were all positives in the PCR assays, if PCR assays were carried out.

**Supplementary Table 2. GT efficiencies for the *GL2* locus using Cas9 driven by various promoters in all-in-one constructs**

| Promoter | T1 | | T2 | | | | | T3 | |
| --- | --- | --- | --- | --- | --- | --- | --- | --- | --- |
|  | PCR | Southern | bulk | | Individual plants | | | bulk | |
|  |  |  | PCR | Southern | Line name | PCR | Southern | PCR | Southern |
| DD45 | 19/28 | 0/8 | 17/27 | N.D. | T2-7 | 18/35 | 0/16 | 0/35 | 0/35 |
|  |  |  |  |  | T2-9 | 20/35 | 0/16 | 0/35 | 0/35 |
|  |  |  |  |  | T2-12 | 13/36 | N.D. | 0/36 | 0/36 |
|  |  |  |  |  | T2-14 | 27/35 | 0/16 | 0/35 | 0/35 |
|  |  |  |  |  | T2-18 | 21/34 | 0/16 | 0/34 | 0/34 |
|  |  |  |  |  | T2-20 | 25/36 | 0/16 | 0/36 | 0/24 |
| YAO | 9/30 | 0/8 | 6/16 | N.D. | T2-33 | N.D. | 0/36 | 0/36 | 0/36 |
|  |  |  |  |  | T2-38 | 20/36 | 0/17 | 0/36 | 0/36 |
|  |  |  |  |  | T2-41 | 3/36 | 0/36 | 0/36 | 0/36 |
| Lat52 | 0/14 | N.D. | 0/14 | N.D. |  |  |  |  |  |
| CDC45 | 16/29 | 0/15 | 11/27 | 0/8 | T2-9 | N.D. | 0/16 | 0/36 | 0/36 |
|  |  |  |  |  | T2-19 | N.D. | 0/16 | 0/36 | 0/36 |
|  |  |  |  |  | T2-20 | N.D. | 0/16 | 0/36 | 0/36 |
|  |  |  |  |  | T2-22 | N.D. | 0/16 | 0/36 | 0/36 |
|  |  |  |  |  | T2-28 | 13/29 | 0/16 | 3/36 | 0/36 |
|  |  |  |  |  | T2-29 | 24/34 | 0/16 | 4/36 | 0/36 |
| CDC45 without sgRNA | 0/53 | N.D. |  |  |  |  |  |  |  |

For Southern analysis, the tested T1 and T2 plants were all positive in the PCR assays, if PCR assays were carried out.

**Supplementary Table 3. GT in different parental backgrounds**

| Construct | Parental line | T2 | | | |
| --- | --- | --- | --- | --- | --- |
|  |  | Bulk | Individual plants | | |
|  |  | PCR | Line name | PCR | Southern |
| *ROS1*-*GFP* | Lat52 | 3/54 | T2-3 | 0/34 | N.D. |
|  |  |  | T2-4 | 0/24 | N.D. |
|  |  |  | T2-5 | 0/20 | N.D. |
| *ROS1*-*Luc* | Lat52 | 4/14 | T2-2 | 7/54 | 0/7 |
|  |  |  | T2-9 | 7/54 | 0/7 |
|  |  |  | T2-11 | 0/54 | N.D. |
|  |  |  | T2-12 | 0/54 | 0/2 |
|  | CDC45 | 0/6 |  |  |  |
|  | YAO | 0/1 |  |  |  |
| *DME*-*GFP* | Lat52 | 0/4 |  |  |  |
|  | CDC45 | 0/1 |  |  |  |
|  | YAO | 1/1 | T2-1 | 0/24 | N.D. |
| *GFP*-*DME* | CDC45 | 5/18 | T2-2 | 9/33 | 0/11 |
|  |  |  | T2-15 | 10/36 | 0/36 |
|  |  |  | T2-16 | 19/36 | 0/36 |
|  |  |  | T2-44 | 16/18 | 0/18 |
|  |  |  | T2-45 | 2/18 | 0/18 |
|  | YAO | 1/8 | T2-9 | 8/18 | N.D. |
| *DME*-*P1633A* | YAO | 0/1 |  |  |  |
|  | CDC45 | 2/6 | T2-21 | 0/31 | N.D. |
|  |  |  | T2-22 | 0/36 | N.D. |
| *DME*-*P1648A* | CDC45 | 1/9 | T2-9 | 0/60 | N.D. |

T2 bulk positive populations were subjected to T2 individual plant analysis by PCR, and the PCR positive individuals and were then analyzed by Southern blotting. Southern blotting was also carried out for some T2 individual plants that were not positive in the PCR assay.

**Supplementary Table 4. Sequences of primers used in this study**

| Primer name | Primer sequence (5’ – 3’) |
| --- | --- |
| sgRNA |  |
| *GL2* | GTCGGAGCATGAAGCCTGCA |
| *ROS1*-*GFP* | CTAACCTCGCCTAATCCGT |
| *GFP*-*DME* | AATTCGAGGGCTGATCCGG |
| *DME*-*GFP* | AACAAAACCTAAAGATGAC |
| *DME*-*P1633A* | TTGGACATGCATTACAATT |
| *DME*-*F1648A* | gcttacCTAGCATAAGCAC |
| Cloning promoters |  |
| Lat52 promoter | TGTCGACATACTCGACTCAGAAGGTA |
|  | GGTACCTTTAAATTGGAATTTTTTTTTTTGGTGTGTGTACTTTTTTT |
| CDC45 promoter | CTCCTGATGATAAAGGTGGGAGTGTC |
|  | TTCCGTGAAATTGAATCACCCAGAAGGC |
| YAO promoter | ACAAATAGAGGTAGGGGGAGAG |
|  | TCTTCTCTCTCTCACTCCCTCTTAG |
| DD45 promoter | AAATGTTCCTCGCTGACGTAAGAAGAC |
|  | AAATGTTCCTCGCTGACGTAAGAAGAC |
| *GFP* and *Luc* donor construction |  |
| *GFP* | ATGGTGAGCAAGGGCGAGGAG |
| With stop-R | TTACTTGTACAGCTCGTCCATGCCGT |
| Without stop-R | CTTGTACAGCTCGTCCATGCCGTG |
| *Luc* | ATGGAAGACGCCAAAAACATAAAGAAAGGC |
|  | TTACACGGCGATCTTTCCGCC |
| *ROS1*-*GFP*-5' arm | GAATTCGTTATTCAACAGGTGAGACGGCTGATTC |
|  | GGCGAGGTTAGCTTGTTGTCC |
| *ROS1*-*GFP*-3' arm | GGCAAGCAAACAAATACAAGCTTATG |
|  | GACAACAATAGAATTCTTTGGTCCGGTTGAACTATC |
| *GFP*-*DME*-5' arm | GCATAGCTGACTCAGTGTTCTCCGG |
|  | AACACACTTGATGAATCACTCCCCC |
| *GFP*-*DME*-3' arm | AATagtAGGGCTGATcctGGGGATAG |
|  | CTGACCCAACTGCTTCTCTTCTAATTGGTGAATTC |
| *DME*-*GFP*-5' arm | GAATTCCAGACAGTTCGAGGAACTCTTCTGGTGAG |
|  | GGTTTTGTTGTTCTTCAATTTGCTCGCAG |
| *DME*-*GFP*-3' arm | AAGAAAGCAAACGCATTGCTTCTCTG |
|  | GAATTCGTGAGTTTTTGCTGGTGCTAACTACTTTGCTC |
| amino acid substitution donor |  |
| *DME*-F | GGAACTTGTATTGATGATATGAAGGTTGACACG |
| *DME*-R | CCGTAGAGTCATTCCAAACTGTTCAATGTTG |
| *P1633A*-substitution | GACATGCATTACAgTTagcTCTACTCTTTGTGC |
| *F1648A*-substitution | CAGACACgctGCtAGTGCaTATGCTAG |
| GT detection |  |
| *GL2*-*GFP* | GATCAGACACATGGAAGCGTACGTTCG |
|  | CTGAACTTGTGGCCGTTCACGTC |
| *ROS1*-*GFP*-5’ | GCAGTTGGAAAAGAGAGAACCTGATGATCC |
|  | CTGAACTTGTGGCCGTTCACGTC |
| *ROS1*-*GFP*-full | ACCTGATGATCCATGTTCTTATTTG |
|  | CCTTGTACAACTCTAGGACTGTT |
| *ROS1*-*GFP*-3’ | ACAACCACTACCTGAGCACC |
|  | TGAAGATCGGAGCTGGTTCC |
| *ROS1*-*Luc*-5’ | GCAGTTGGAAAAGAGAGAACCTGATGATCC |
|  | CCAGGAACCAGGGCGTATCTCTT |
| *GFP*-*DME*-5’ | ACTTAAGCAACAACGTCCTCGTGAATATAATTTGG |
|  | CTGAACTTGTGGCCGTTCACGTC |
| *DME*-*GFP*-5’ | GCTTTGACGAGACTTGTTCTGAGTGTAACAG |
|  | CTGAACTTGTGGCCGTTCACGTC |
| *DME*-substitution | ATGCGTGCGACTCTTAACAC |
|  | TGCTCCACTTGCTAGGGATT |
| Genotyping & RT-PCR |  |
| Cas9 | CAGCCGACAAGAAGTACAGC |
|  | ATGGTGGGGTACTTCTCGTG |
| bar | GAAGTCCAGCTGCCAGAAAC |
|  | CCAACCACGTCTTCAAAGCA |

| *Actin* | GGTTCGTGGTGGTGAGTTTG |
| --- | --- |
|  | GCTGCATTGTCACCCGATAC |
| qPCR |  |
| *ROS1*-qRT | ACCAAACGAAGGGAACAGAGA |
|  | ACAGTCCTAGAGTTGTACAAGGT |
| *Actin*-qRT | TCCATCGATTGTTCACAGGA |
|  | TCACCACCACGAACCAGATA |
| At1g26400-BstUI-qChop | TGACCTGCATAGGCTATAACACA |
|  | ATTGGAATCAATCCGAGTGG |
| At1g03890-BstUI-qChop | CGTGCATTATTTTGGCAGTAACA |
|  | ATGCGTCCGGATTTCAGTAT |
| bisulfite sequencing |  |
| *ROS1*-*GFP*-5'-bis | GtAAATGGTATGtTTTGTGAtGAGGAGAtTTG |
|  | CCAAAAGCAAaCCTaTATCTTCTCAaTTaATAAACC |
| *ROS1*-*GFP*-3'-bis | GTTttAATtTAGTTAATGTAAGAAAGTGAAAAtGTAAAG |
|  | CATTACAACAATCAaTAACCCTAAaTTACAATCTA |
